# Draft Genome Sequences of *Brucella suis* Biovar 4 Strain NCTC 10385*, Brucella ceti* Strain NCTC 12891^T^*, Brucella inopinata* Strain CAMP 6436^T^, and *Brucella neotomae* Strain ATCC 23459^T^

**DOI:** 10.1128/genomeA.00783-14

**Published:** 2014-10-02

**Authors:** Tara Wahab, Sevinc Ferrari, Martina Lindberg, Stina Bäckman, Rene Kaden

**Affiliations:** a Swedish Forum for Biopreparedness Diagnostics, Umeå, Uppsala and Solna, Sweden; b Public Health Agency of Sweden, Solna, Sweden; c National Veterinary Institute, Uppsala, Sweden; d National Food Agency, Uppsala, Sweden; e Swedish Defence Research Agency, Umeå, Sweden; f Uppsala University, Department of Medical Sciences, Uppsala, Sweden

## Abstract

With the aim of developing quantitative PCR methods for the detection and differentiation of *Brucella* species, the genomes of *Brucella ceti*, *Brucella inopinata, Brucella netotomae*, and *Brucella suis* biovar 4 were sequenced and analyzed.

## GENOME ANNOUNCEMENT

All species of the genus *Brucella* are facultative intracellular bacteria, and many species have a high zoonotic potential. *Brucella ceti* was detected in whales, dolphins, seals, and sea lions (1). Human infections are described but might be sporadic (2, 3, 4). *Brucella inopinata* was detected in only one human isolate. Currently, the pathogenicity is unclear. *Brucella netotomae* seems to be host-specific and was not reported to cause human infections (5), whereas *Brucella suis* biovar 4 causes brucellosis in reindeer, caribou wolves, bears, foxes, humans, rodents, and musk ox (6, 7).

For the development of a new genus-specific real-time PCR, the species that are described above had to be sequenced due to the lack of those sequences in the publicly available databases at the start of the project.

In preparation of the sequencing, 1 µg extracted DNA per strain was used for Ion Torrent library construction on the AB Library BuilderTMsystem using the Ion XpressTM Plus Fragment library kit. Lab Chip XTTM was used for quality control of the libraries. The libraries were enriched on beads using the OneTouchTM 2 system. The final libraries were sequenced in multiplex on an Ion 318 chip V2 using 2 mL of control ISPs.

The data were demultiplexed in Torrent SuiteTM and analyzed in CLC Genomics Workbench version 6.5.1. The raw read length distribution was 262 bp, and the average read length was 207 bp. The coverage for the sequences of *B. suis*, *B. ceti*, *B. inopinata*, and *B. neotomae* was 60×.

The four datasets were *de novo* assembled using standard settings in CLC Genomics Workbench, and the resulting contigs were annotated using the BLAST annotation tool. The main contig information is summarized in Table 1.

**TABLE 1:** Statistics for the draft genomes

Genome	Accession number	*N*_50_ contig length (bp)	Maximum contig length (bp)	No. of contigs of >500 bp
*B. suis*	AZBG00000000	32,219	115,058	218
*B. ceti*	AZBH00000000	29,208	94,523	206
*B. inopinata*	AZBI00000000	29,979	166,243	212
*B. neotomae*	AZBJ00000000	34,109	97,858	177

The CG content determination was realized with Gegenees version 2.0 (8), which gave the following results: *Brucella ceti*, 57.28%; *Brucella inopinata*, 57.25%; *Brucella suis* BV4, 57.03%; and *Brucella neotomae*, 57.23%. Tandem repeats were detected using the Tandem Repeats Finder (9). While the genome of *B. suis* BV4 contained 84 tandem repeats with a maximal repeat number of 4, in the genomes of *B. ceti*, *B. neotomae*, and *B. inopinata*, 55, 49, and 63 tandem repeats with frequencies of 7, 10, and 7, respectively, were detected.

### Nucleotide sequence accession numbers.

The sequences belong to NCBI Bioproject PRJNA230241 and were deposited at DDBJ/EMBL/GenBank under the accession numbers AZBG00000000 (*Brucella suis* BV4 NCTC 10385), AZBH00000000 (*Brucella ceti* NCTC 12891^T^), AZBI00000000 (*Brucella inopinata* CAMP 6436^T^), and AZBJ00000000 (*Brucella neotomae* ATCC 23459^T^).
